# Combined treatment with antioxidants and immunosuppressants on cytokine release by human peripheral blood mononuclear cells - chemically injured keratocyte reaction

**Published:** 2011-10-15

**Authors:** Kayoung Yi, Tae Young Chung, Joon Young Hyon, Jae Woong Koh, Won Ryang Wee, Young Joo Shin

**Affiliations:** 1 Department of Ophthalmology, Hallym University College of Medicine, Seoul, Republic of Korea; 2 Department of Ophthalmology, Samsung Medical Center, Sungkyunkwan University School of Medicine, Seoul, Republic of Korea; 3 Department of Ophthalmology, Seoul National University College of Medicine, Seoul, Republic of Korea; 4 Department of Ophthalmology, Chosun University School of Medicine, Gwangju, Republic of Korea

## Abstract

**Purpose:**

To investigate the effect of antioxidants and immunosuppresants on mixed peripheral blood mononuclear cells (PBMC) - chemically injured keratocytes reaction (MLKR).

**Methods:**

The PBMC stimulation assay was performed using chemically injured keratocytes treated with 0.05 N NaOH for 90 s (MLKR). MLKR were treated with various drugs including rapamycin, dexamethasone, mycophenoleic acid (MPA), alpha lipoic acid (ALA), and N-acetyl cysteine (NAC). Matrix metalloprotease-9 (MMP-9), transforming growth factor–beta 1 (TGF-β1), interleukin-6 (IL-6), and macrophage migration inhibitory factor (MIF) secretion profiles of activated PBMCs stimulated by NaOH-treated keratocytes were determined by ELISA.

**Results:**

Anti-oxidants as well as immunosuppressants suppressed PBMC proliferation. MMP-9 levels were lower in antioxidants group. IL-6 levels decreased in dexamethasone group and anti-oxidants group. Combination of immunosuppressants and antioxidants suppressed more PBMC proliferation except for rapamycin + ALA group, suppressed MMP-9 production except for MPA + ALA group, decreased IL-6 levels and increased MIF levels except for rapamycin + ALA group. TGF-β1 levels were elevated in rapamycin group and rapamycin + ALA group.

**Conclusions:**

Cytokine production was different depending on combination of drugs.Our results suggest that the different drugs should be selected for treatment according to the phases of corneal chemical burn.

## Introduction

Corneal chemical burn can induce a devastating and permanent damage to ocular surface resulting in corneal blindness [1]. Corneal chemical burn injuries can induce a large extent of cell death [2]. Especially, exposure to alkali agent may cause extensive damage to ocular tissues because alkali can progress rapidly and penetrate into deep tissues [3]. Although there have been many studies about treatment of chronic ocular damages including amniotic membrane transplantation, oral mucosal transplantation and limbal transplantation [3,4], suppression of acute and chronic inflammation induced by chemical burn still has been challenging.

A variety of medical therapies including topical and systemic drugs have been investigated to control inflammation and promote ocular surface healing [5,6]. After reactive oxygen species (ROS) has been reported to be able to induce inflammation [7,8], there have been many studies to report the effect of anti-oxidants on inflammation [2,7-10]. However, the effect of combination of immunosuppressants and anti-oxidants on corneal chemical burn has not been studied. In this study, we investigated the effect of antioxidants and immunosuppresants on mixed peripheral blood mononuclear cells (PBMC) - chemically injured keratocytes reaction (MLKR).

## Methods

This study was performed according to the tenets of the Declaration of Helsinki and was reviewed and approved by the institutional review board/ethics committee of Hallym University Medical Center, Seoul, Republic of Korea. Human corneal cells, including human corneal keratocytes and epithelial cells, were obtained from discarded corneal–scleral rings after penetrating keratoplasty. These tissues were stored in Optisol^™^-GS (Bausch and Lomb Inc., Rochester, NY) at 4 °C until processed for culture.

### Human corneal keratocyte culture

Descemet's membrane and epithelium were removed using forceps and an ophthalmic knife, and stroma was minced under laminar flow. Mid-stroma and posterior stroma explants were then suspended in culture medium and cultured in 24-well plates [11-13]. The corneal stroma was sliced into quarters and digested overnight with 2.0 mg/ml collagenase (Roche, Basel, Switzerland) and 0.5 mg/ml hyaluronidase (Worthington Biochemicals, Lakewood, NJ) in DMEM at 37 °C. Isolated cells were washed in DMEM and cultured in DMEM/F12 supplemented with 10% fetal bovine serum (FBS; Gibco-Invitrogen, Grand Island, NY). The cells were cultured on tissue culture-treated plastic at 4×10^4^ cells/cm^2^.

### PBMC isolation

Heparinized fresh whole blood (10 IU heparin/ml) was diluted 1:2 with PBS solution. The PBMC fraction was obtained by Ficoll-Hypaque centrifugation. The cells were then washed in PBS before culture. The PBMCs were cultured for 24 h at 37 °C at a density of 1×10^6^ cells/well in Roswell Park Memorial Institute (RPMI) medium supplemented with 5% (vol/vol) fetal calf serum. The viability of PBMCs was measured by trypan blue dye exclusion and was consistently greater than 98%. The cells were then suspended in RPMI-1640 (Invitrogen-Life Technologies).

### PBMC stimulation assay

The PBMC stimulation assay was performed to determine immunoreactivity as previously described [14,15]. In this investigation, mitomycin C and 0.05N NaOH–treated keratocytes (5×10^5^/ml) were used as the stimulators. They were incubated with 25 µg/ml mitomycin C for 30 min in a 5% CO_2_ humidified incubator [16]. Residual mitomycin C was removed by repeated washing (3 times) with RPMI-1640 containing 10% FBS. Then keratocytes were treated with 0.05N NaOH for 90 s and washed three times with RPMI-1640 containing 10% FBS. Responding cells (1×10^6^/well) and stimulating cells were cocultured in 200 μl of RPMI-1640 in 96-well plates. The cocultures were incubated with immunosuppressants including 25 nM rapamycin, 10 µM dexamethasone and 10 µM mycophenoleic acid (MPA), or anti-oxidants including 50 mM alpha lipoic acid (ALA) and 15 mM N-acetylcysteine (NAC). The plates were incubated for 2 days, and PBMC proliferation was measured using a commercial bromodeoxyuridine (BrdU) proliferation assay kit (Roche Diagnostics), according to the manufacturer’s protocol. One hundred microliters of each cell suspension with 1×10^6^ PBMC/ml was added to 5 wells of flat bottomed 96-well plates. Unstimulated PBMCs served as the negative control, and phytohemagglutinin (PHA, 5 µg/ml; Sigma Chemical Co., St. Louis, MO)-treated PBMCs as the positive control. The optical density was measured at 450 nm with an ELISA reader [17]. The results are expressed as mean (SD). Proliferation rates were calculated as the percentage increase in the number of cells between PHA-stimulated and unstimulated cultures, after subtraction of the corresponding blanks.

### Enzyme-linked immunosorbent assay (ELISA)

The supernatant was collected after centrifugation at 300× g for 10 min, and frozen at −70 °C until used for measurement of cytokine levels. The concentration of interleukin-6 (IL-6), macrophage migration inhibitory factor (MIF), transforming growth factor - β1 (TGF- β1), and matrix metalloprotease-9 (MMP-9) in PBMC culture medium was assayed using commercial human IL-6, MIF, TGF-β, and MMP-9 ELISA kits (R&D Systems, Minneapolis, MN), according to the manufacturer’s protocols. The sensitivity limit of each ELISA was about 500 pg/ml. In brief, anti-human MMP-9, or TGF-β1, or MIF, or IL-6 antibody was added to each well of a 96-well microtiter plate and incubated overnight at room temperature. Next day, each well was washed with washing buffer. All wells were then filled with blocking buffer (pH 7.4) containing 1% BSA and left for 1 h at room temperature. One hundred microliters of the standard dilutions of human MMP-9, or TGF-β1, or MIF, or IL-6 of experimental samples were dispensed into a 96-well microtiter plate coated with the appropriate polyclonal antibody. The plate was sealed and incubated at room temperature (RT) for 2 h. After this, the plates were washed 4 times, and 100 μl of goat anti-human MMP-9, or TGF-β1, or MIF, or IL-6 conjugated to horseradish peroxidase was added to each well and allowed to incubate at RT for 2 h. One-hundred-microliter aliquots of the color reagent 3,3′,5,5′-tetramethylbenzidine (TMB) were then applied for 20 min to develop a blue color, and the reaction was stopped by adding 50 μl of 1 M H_2_SO_4_. The absorbance was measured at a wavelength of 450 nm by using an automatic plate reader with a reference wavelength of 570 nm.

## Results

### Culture of human keratocytes

**Figure 1:**
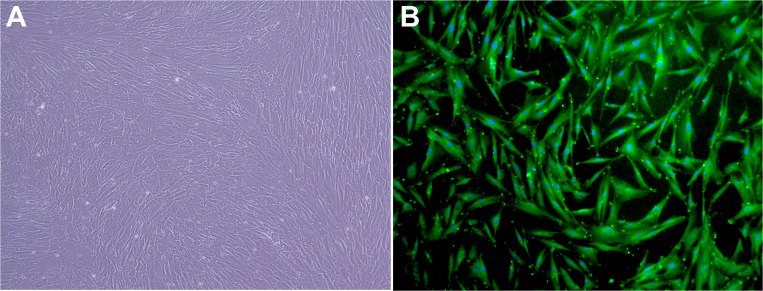
Identification of keratocytes. Inverted phase-contrast images (**A**) and immune florescent vimentin staining (**B**) of human keratocytes. Keratocytes were observed growing parallel to the etched lines. Immunofluorescence with anti-vimentin antibody, a general marker of keratocyte, showed the morphology. The nuclei were stained with Hoechst 33342 (blue). Magnification, 200×.

Cultured keratocytes were observed growing parallel to the etched lines (Figure 1A). Immunofluorescence with anti-vimentin antibody, a general marker of keratocyte, showed the morphology (Figure 1B). The nuclei were stained with Hoechst 33342.

### PBMC stimulation assay

**Figure 2:**
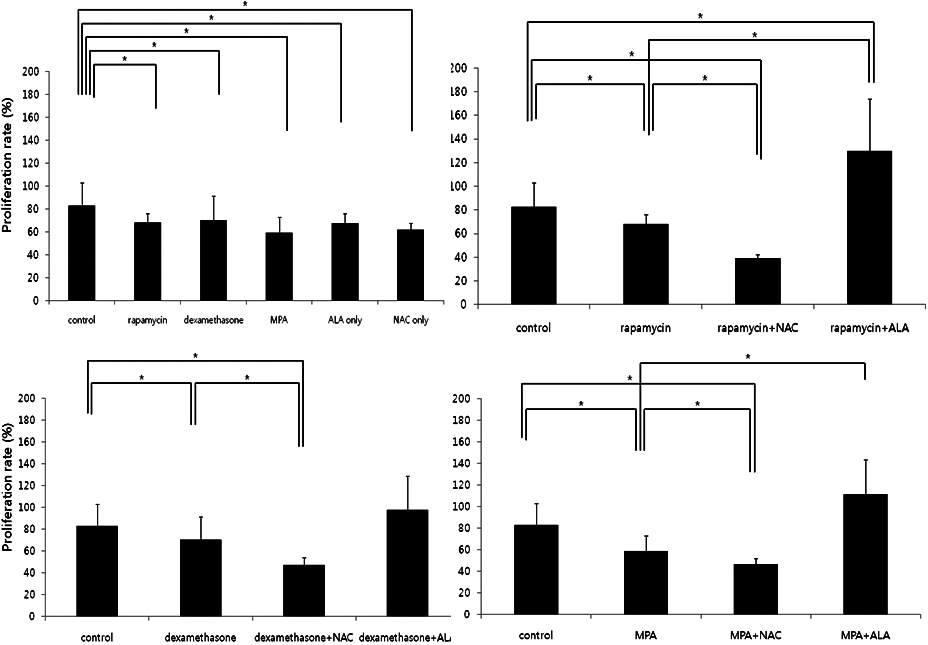
PBMC stimulation test. NaOH-treated human keratocytes served as the stimulators. Anti-oxidants as well as immunosuppressants suppressed the PBMC proliferation (Top left). The combination of immunosuppressants and NAC was more effective in suppression of PBMC proliferation while the combination of immunosuppressants and ALA increased PBMC proliferation except for dexamethasone.

NaOH-treated human keratocytes served as the stimulators. Anti-oxidants as well as immunosuppressants suppressed the PBMC proliferation (p=0.016, 0.027, 0.028, 0.016, and 0.016, respectively, Mann–Whitney U test; Figure 2). The combination of immunosuppressants and NAC was more effective in suppression of PBMC proliferation (p=0.009, 0.009, and 0.009, respectively, Mann–Whitney U test) while the combination of rapamycin and ALA increased PBMC proliferation (p=0.009, Mann–Whitney U test). Dexamethasone + ALA group and MPA + ALA group did not show any difference (Figure 2).

### MMP-9 secretion profiles of activated PBMCs

**Figure 3:**
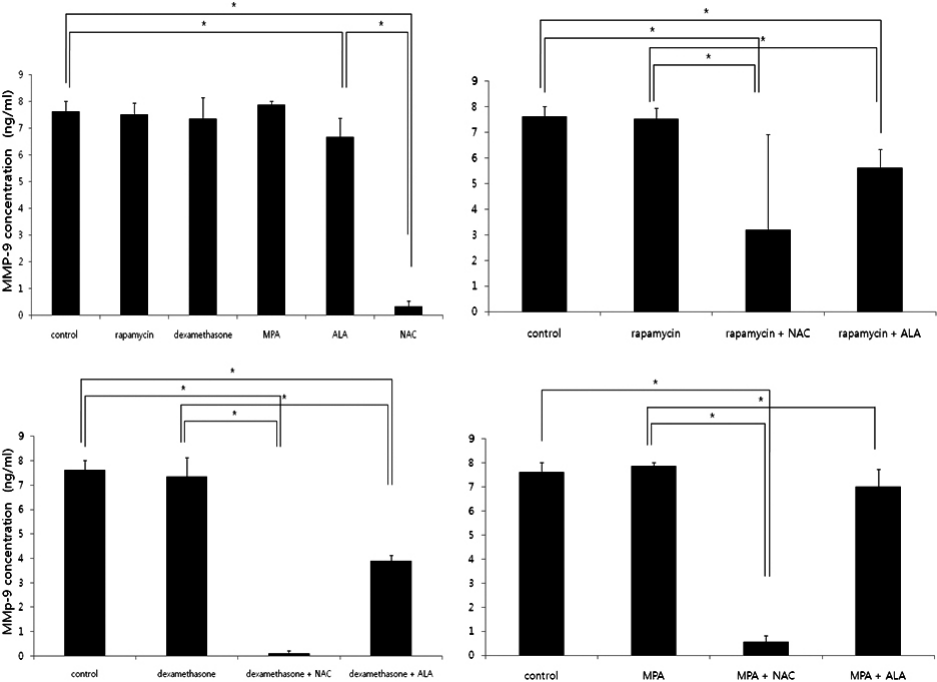
MMP-9 levels measured by ELISA. MMP-9 levels were lower in ALA group and NAC group (Top left). The combination of immunosuppressants and antioxidants was more effective in suppression of MMP-9 production except for the combination of MPA and ALA.

MMP-9 levels were lower in ALA group and NAC group (p=0.028 and 0.009, respectively, Mann–Whitney U test). The combination of immunosuppressants and antioxidants was more effective in suppression of MMP-9 production except for the combination of MPA and ALA (p=0.028 in rapamycin + NAC group, 0.009 in rapamycin + ALA group, 0.009 in dexamethasone + NAC, 0.009 in dexamethasone + ALA group and 0.009 in MPA + NAC group, respectively, Mann–Whitney U test; Figure 3).

### IL-6 secretion profiles of activated PBMCs

**Figure 4:**
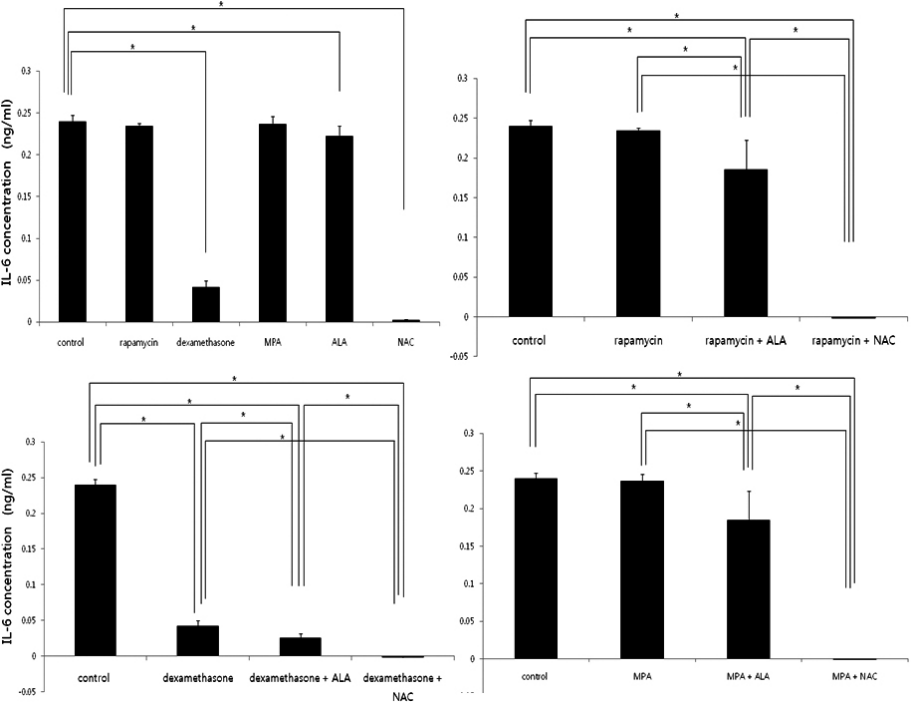
IL-6 levels measured by ELISA. IL-6 levels decreased in treatment with dexamethasone and anti-oxidants (Top left). The combination of immunosuppressants and antioxidants are more effective to suppress the production of IL-6 .

IL-6 levels decreased in treatment with dexamethasone or anti-oxidants (p=0.029, 0.043 and 0.021, respectively, Mann–Whitney U test; Figure 4). A combination of immunosuppressants and antioxidants are more effective to suppress the production of IL-6 (p=0.034 in rapamycin + ALA group, 0.020 in rapamycin + NAC group, 0.021 in dexamethasone + ALA group, 0.021 in dexamethasone + NAC group, 0.019 in MPA + ALA group, and 0.021 in MPA + NAC group, respectively, Mann–Whitney U test; Figure 4).

### MIF secretion profiles of activated PBMCs

**Figure 5:**
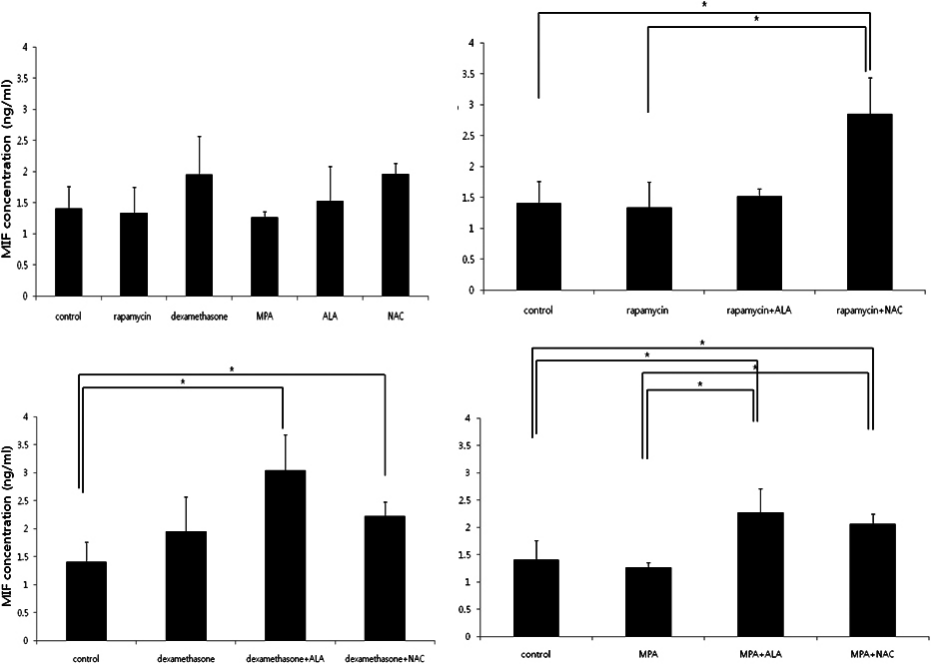
MIF levels measured by ELISA. MIF levels were not different in treatment with each drug (Top left). MIF levels increased in MLKR treated with the combination of immunosuppressants and antioxidants except for the combination of rapamycin and ALA.

MIF levels were not different in treatment with each drug (Figure 5). MIF levels were elevated in treatment with combination of immunosuppressants and NAC (p=0.034, 0.034, and 0.034, respectively, Mann–Whitney U test; Figure 5). MIF levels in rapamycin + NAC group were higher compared to rapamycin group (p=0.021, Mann–Whitney U test) and those in MPA + ALA group and MPA + NAC group were higher compared to MPA group (p=0.021 and 0.021, respectively, Mann–Whitney U test; Figure 5).

### TGF-β1 secretion profiles of activated PBMCs

**Figure 6:**
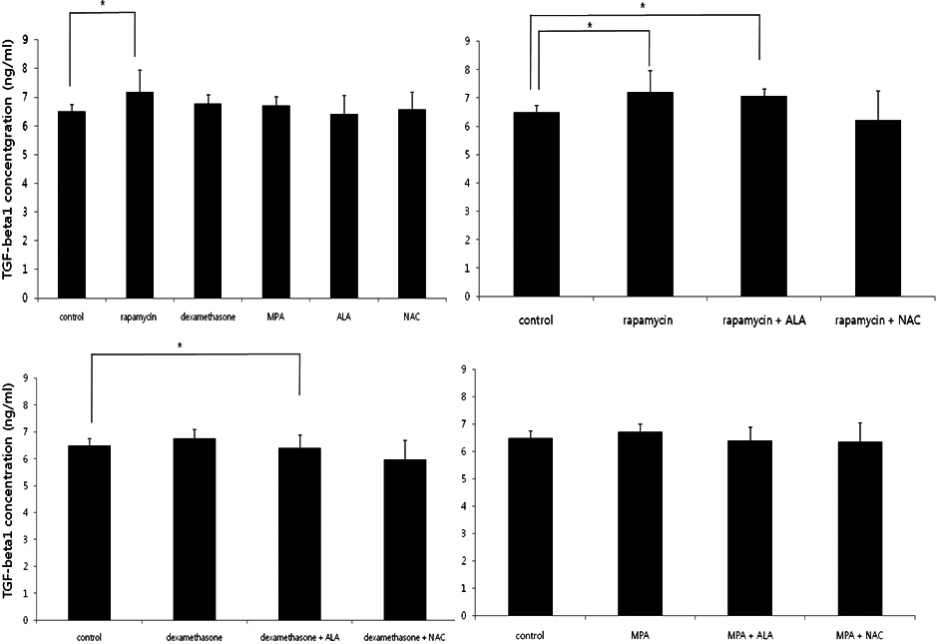
TGF-β1 levels measured by ELISA. TGF-β1 levels were elevated in rapamycin group, rapamycin + ALA group.

TGF-β1 levels were elevated in rapamycin group, rapamycin + ALA group and dexamethasone + ALA group (p=0.047, 0.009, and 0.047, respectively, Mann–Whitney U test; Figure 6).

## Discussion

Corneal chemical burn injury can induce inflammation [4]. Alkali agents saponify the cellular membrane and induce cell death [3]. Cellular membrane damage is the cause of inflammation in necrosis [5]. Necrosis induces inflammation while apoptosis doesn’t [18]. Thus, chemically injured keratocyte may play an important role in inducing inflammation in corneal chemical burn. Interactions between chemical-burned keratocyte and PBMC may play an essential role in chemical burn because keratocytes are the major cellular components of cornea [9]. In addition, it has been reported that keratocyte have a functional plasticity of resident stromal cells and can become macrophage-like as a first response to injury or infection of the cornea [19].

In this study, we found that anti-oxidants as well as immunosuppressants suppress the PBMC proliferation. The combination of immunosuppressants and NAC was more effective in suppression of PBMC proliferation. It was reported that low concentrations of ROS can stimulate cellular proliferation as a second messenger [10,17]. ROS are implicated in the regulation of several cellular processes depending on their intracellular levels. Although high levels of ROS are toxic to the cells [1,5], low levels of ROS have various physiologic roles for appropriate signal transduction, kinase activation, and biologic responses associated with receptor signaling [6]. In addition, ROS has been described to modulate the inflammation [7] and promote inflammatory cytokines [6]. Thus, antioxidants can have a potential to suppress inflammation. On the other hand, the combination of immunosuppressants and ALA increased PBMC proliferation except for the combination of dexamethasone and ALA. Although it has been reported that ALA alone inhibits air way inflammation [20,21], we found the combination of immunosuppressants and ALA may increase inflammation on the contrary . Further molecular-based studies are needed to clarify these results.

MMP-9 is a collagenase IV produced by PBMCs [21,22]. The production of MMPs by PBMC are triggered by chemokines and cytokines produced by other inflammatory cells at the site of inflammation [23]. MMP-9 is involved in degradation of extracellular matrix [24]. Corneal stroma can be thinned progressively by MMP-9 upregulation in corneal chemical burn [25]. Suppression of MMP-9 is important to prevent cornea from thinning. In this study, MMP-9 levels were lower in ALA group and NAC group. The combination of immunosuppressants and antioxidants was more effective in suppression of MMP-9 production except for the combination of MPA and ALA.

IL-6 is a proinflammatory cytokine produced by PBMC [26]. In this study, IL-6 levels decreased in treatment with dexamethasone and anti-oxidants. The combination of immunosuppressants and antioxidants are more effective to suppress the production of IL-6. MIF levels were not significantly different in treatment with each drug. MIF levels increased in MLKR treated with the combination of immunosuppressants and antioxidants except for the combination of rapamycin and ALA. MIF has been described to be an innate immunity molecule leading to induction of proinflammatory activities [27]. MIF was originally described as a regulator of macrophage responses [27]. It directly or indirectly promotes expression of a variety of pro-inflammatory cytokines including IL-6, TNF-alpha, IL-8 [28-30], MMP-9 [31], and TGF-β1 [32,33]. MIF levels were elevated in the groups which showed lower PBMC proliferation rates and lower IL-6 levels in this study. MIF might be elevated as a master regulator of inflammation [8,29] in response to suppression of inflammation. TGF-β1 is a multifunctional cytokine that participates in a wide range of biologic events, including inflammation and wound repair [32]. TGF-β1 induces synthesis and accumulation of extracellular matrix protein and has been implicated as the potent and key mediator of fibrogenesis [33]. TGF-β1 has been reported to be induced by MIF in IgA nephropathy [33] while reduced TGF-β1 and increased MIF has been described in severe malaria [34]. In this study, TGF-β1 levels were elevated only in rapamycin group and rapamycin + ALA group. TGF-β1 plays an essential role in corneal chemical burn via inducing inflammation and repairing wound [35]. TGF- β1inducing transdifferentiation from keratocyte to myofibroblast contribute these processes [35].

In conclusion, anti-oxidants as well as immunosuppressants suppressed the PBMC proliferation induced by chemically injured keratocyte. The combination of immunosuppressants and anti-oxidants had a synergic effect on MLKR. Cytokine production was different depending on the combination of drugs. Our results suggest that the different drugs should be selected for treatment according to the phases of corneal chemical burn. Further study is necessary to investigate the interaction between immunosuppressants and antioxidants.
